# Success of hydrocone (TORIS-K) soft contact lens for keratoconus and traumatic keratopathy

**DOI:** 10.12669/pjms.314.6125

**Published:** 2015

**Authors:** Ahmet Altun, Sevda Aydin Kurna, Tomris Sengor, Gulengul Altun, Osman Okan Olcaysu, Mert Hakan Simsek

**Affiliations:** 1 Ahmet Altun, Fatih Sultan Mehmet Education and Research Hospital, Clinic of Ophthalmology, Istanbul, Turkey; 2 Sevda Aydin Kurna, Fatih Sultan Mehmet Education and Research Hospital, Clinic of Ophthalmology, Istanbul, Turkey; 3 Tomris Sengor, Department of Ophthalmology, Istanbul Bilim University, Istanbul, Turkey; 4 Gulengul Altun, Department of Pediatrics, Yeditepe University, Istanbul, Turkey; 5 Osman Okan Olcaysu, Erzurum Region Education and Research Hospital, Clinic of Ophthalmology, Erzurum, Turkey; 6 Mert Hakan Simsek, Fatih Sultan Mehmet Education and Research Hospital, Clinic of Ophthalmology, Istanbul, Turkey

**Keywords:** Keratoconus, Hydrocone contact lens, Astigmatism

## Abstract

**Objective:**

To present success of Toris-K contact lenses in keratoconus and traumatic keratopathy with irregular corneal surface.

**Methods:**

Toris-K contact lenses were used to treat 7 eyes of 4 patients with traumatic keratopathy (Case 1) or keratoconus (Case 2, Case 3, and Case 4). All cases had a complete eye examination before the contact lens application. The case with traumatic keratopathy was a 32-year-old male who had corneal penetrating injury due to hobnail strike 23 months ago. The other 3 keratoconus cases were females at the age of 14, 16 and 22 years old. They had high myopia and irregular astigmatism due to keratoconus. All patients refused using rigid gas permeable contact lens because of intolerance. Toris-K contact lenses were fitted on all eyes. All patients were followed-up for 28 months with a complete ophthalmic examination and corneal topography every two months.

**Results:**

Improvement of BCVA of the cases was remarkable. All cases were comfortable with their Toris-K contact lenses for 28 months. There was no significant distortion on the lenses during follow-up period.

**Conclusion:**

Toris-K lenses may be an effective alternative treatment option for the patients with keratoconus and traumatic keratopathy, especially who cannot tolerate rigid gas permeable contact lenses.

## INTRODUCTION

Keratoconus is a non-inflammatory disease of the cornea characterized by thinning of the corneal stroma that may lead to irregular astigmatism and decrease in visual acuity. It typically commences at puberty and progresses to the mid 30s.^1^-^4^ The main goal of treatment of keratoconus has changed over the last few years from that focused mainly on improvement of visual acuity to that focused on the prevention of progression of the disease. Current treatment options for keratoconus are spectacles, contact lenses, corneal collagen cross-linking, intracorneal ring segments, intraocular lenses, excimer laser and corneal transplantation.^5^-^7^

In the early stages of keratoconus, spectacles are usually the first option, especially for patients who achieve high visual acuity, but spectacles may not have satisfying success for correcting irregular astigmatism, in which rigid gas permeable contact lenses (RGPCL) usually provide better correction. RGPCL are usually the first choice for many patients because of providing good visual acuity. The main difficulty for this application is finding the best suitable contact lens for irregular corneal surface, which is sometimes impossible. Irregular corneal surface is usually a component of keratoconus, but it might develop after penetrating injuries of the eye. To help these kinds of cases, many techniques and different contact lens designs have been described in recent years.

Another problem of the RGPCL might be lens intolerance due to the sensation of foreign body and the redness of the eye. Hybrid and piggyback contact lenses are some available options to solve this problem. In this study, we would like to present a novel soft contact lens, as called Toris-K, that might be an effective and tolerable treatment option for the patients with irregular corneal surface due to keratoconus and traumatic keratopathy.

## METHODS

Seven eyes of 4 patients with keratoconus or traumatic keratopathy treated with Toris-K contact lenses (SwissLens SA, Prilly, Switzerland) were enrolled in to this study. Each patient underwent a detailed clinical evaluation that included recording of medical history, Snellen visual acuity testing, slit-lamp biomicroscopy, Goldman applanation tonometry, and cornea topography. After explaining the aim of the study, informed consent was obtained in accordance with the Helsinki Declaration prior to the procedures. The Institutional Review Board approved our review of the patients’ data.

**Case 1** was a 32-year-old male who had laser-assisted in situ keratomileusis in both eyes 6 years ago and corneal penetrating injury to the right eye 23 months ago. Fig-I After three months the primary corneal suturing, cataract extraction was made with phacoemulsification surgery in another ophthalmology clinic. After removing the corneal sutures, his best corrected visual acuity (BCVA) of the right eye was 20/100 with spectacles. RGPCL applications were unsuccessful because of advanced corneal surface. Fig-II BCVA of the left eye was 20/20 with glasses (-0,50 spherical -0,25 cylindrical with the axis of 15). Dilated fundus examination was unremarkable bilaterally. Intraocular pressure was within normal limits in the right (digitally) and left eye (Goldmann applanation tonometry) respectively.

**Fig.1:**
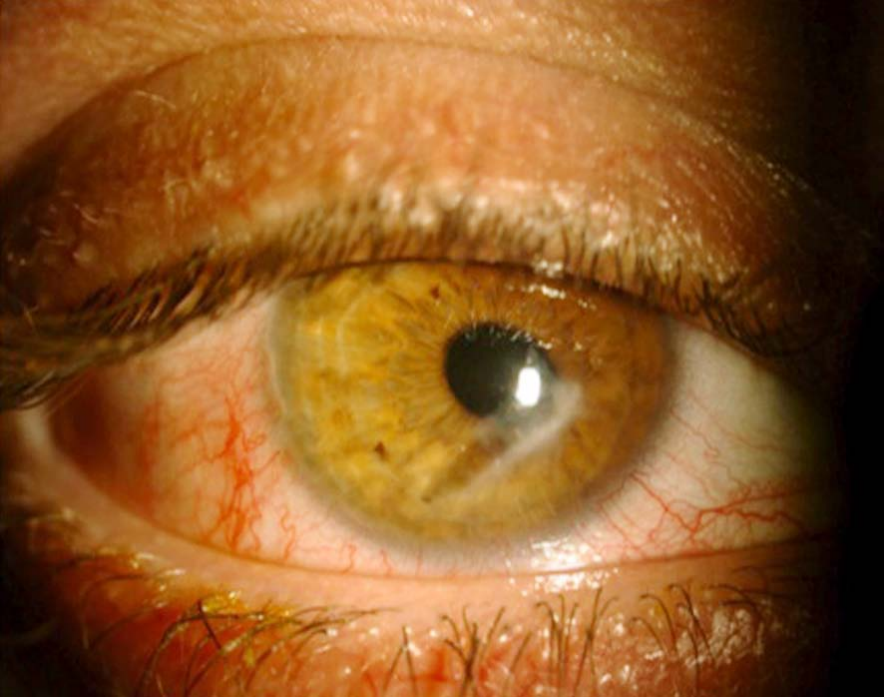
Paracentral corneal scar in the right eye of the Case 1 due to corneal penetrating injury.

**Fig.2:**
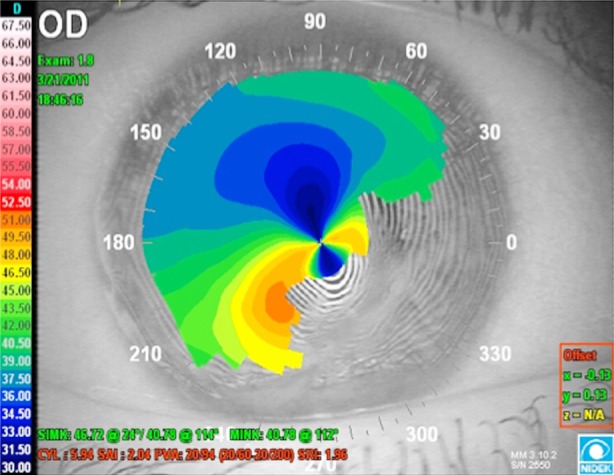
Irregular corneal surface due to scar in the right eye of the Case 1.

**Case 2, Case 3, and Case 4** were the patients with keratoconus at the age of 14, 16 and 22 years. They had high myopia and irregular astigmatism and were following up at out patient clinic. Table-I demonstrates the refractive errors of the cases. They were all reducing to use RGPCL because of intolerance. They had the diagnosis of keratoconus for 11, 20 and 12 months, respectively. BCVA of the right and left eyes with glasses were 20/100 and 20/200 in Case 2, 20/80 and 20/100 in Case 3, and 20/60 and 20/80 in Case 4, respectively.

**Table-I:** Refractive errors of the keratoconus cases.

	Right Eye		Left Eye	
	Spherical	Cylindrical (X: Axis)	Spherical	Cylindrical (X: Axis)
Case 2	-6,50	-2,50 X70	-8,50	-2,25 X60
Case 3	-5,25	-3,00 X50	-6,00	-3,50 X65
Case 4	-5,50	-2,00 X75	-6,25	-3,50 X60

Toris-K contact lenses were fitted on all eyes. All cases were followed-up for 28 months with a complete ophthalmic examination and corneal topography every two months. To find the most appropriate option, at least three different base curves (BC) were tried for each case. Refractive measurements were retaken 45 minutes after the application. At the end of the examination the patients were also asked about their preferences and the comfort.

## RESULTS

BCVA of the Case one improved to 20/20 in the right eye. BCVA of the patients with keratoconus in the right and left eye improved to 20/20 bilaterally in Case 3 and Case 4. BCVA of Case 2 was improved to 20/40 and 20/60 in the right and left eye, respectively. The mean topographic keratometry values of the patients with keratoconus were 51.6 (range: 48.4 and 54.6).

After application, all cases were comfortable with their Toris-K contact lenses except Case 2, who had mild conjunctival hyperemia in the beginning that regressed after using preservative-free artificial eyedrops containing polyvinyl alcohol and povidone (Refresh, Allergan, Ireland), 5 times a day. All cases are still using their prescribed Toris-K contact lenses approximately for 28 months without any intolerance, sensation of foreign body or red eye. There was no significant distortion of the lenses during follow-up period.

## DISCUSSION

Contact lenses are effective and minimal invasive treatment option for advanced keratoconus and irregular corneal surfaces, wherein keratoplasty was thought to be the only treatment option before. In cases where the use of glasses does not provide sufficient visual acuity, contact lenses significantly improves the visual acuity. Smiddy et al have shown that approximately 70% patients who present for surgical consideration with keratoplasty for keratoconus can be maintained successfully on contact lenses.^8^ Providing a contact lens, which is comfortable and ensures an improvement in visual acuity, reduces the need for surgical intervention. Contact lenses have been reported to reduce astigmatism, thus enhancing the quality of vision.^9^ That is why; several manufacturers have developed special lenses for keratoconus patients.

RGPCL improve visual acuity not only by their refractive power but also by providing a regular corneal surface. The main difficulty of this application is usually the difficulty of finding the best suitable contact lens for the irregular corneal surface. Paracentral touch or frequent lens dislocation are some common problems of RGPCL, especially during dusty, windy conditions or at the gym. Because of their rigid structure, some patients may give up using RGPCL because of intolerance due to irritation.

For these reasons different contact lens options emerged, for example hybrid and piggyback contact lenses. Hybrid lenses developed to bring the high-quality vision of RGPCL and the comfort of soft lenses.^10^ The Piggyback lens may be a good option in the advanced stages in order to provide convenience and comfort with RGPCL intolerance in cases of keratoconus.^11^

**Fig.3:**
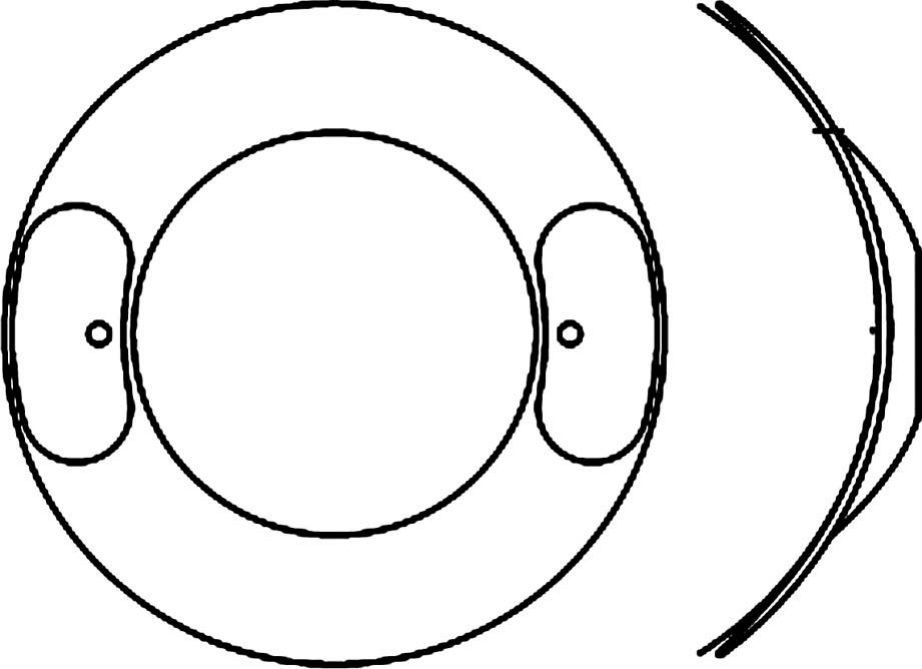
Toris-K contact lens has a front toric surface and dynamic (with bumps) stabilization.

Toris-K contact lens (SwissLens SA, Prilly, Switzerland), that we have used on our patients, is a novel silicone-hydrogel soft contact lens with the options of diameter between 12.00 mm and 17.00 mm, base curve between 7.20 mm and 10.80 mm, spheric power between -40.00 D and +40.00 D, cylinderic power between -0.25 D and -8.00 D, axis options between 0° and 180°, central thickness between 0.35 mm and 0.59 mm, and optical zone between 5.00 mm and 7.50 mm (Table-II). The contact lens has a front toric surface and dynamic (with bumps) stabilization. It consists of 74% water and a thickened centre with a tapering to the periphery. A schematic design is shown in Fig.3. All our cases were very comfortable with Toris-K contact lenses. Only one patient had conjunctival hyperemia for a few days in the beginning that was probably due to unsuitable usage. We have followed up the patients for 28 months. In this period, there was no progression of keratoconus according to their keratometry and refractive parameters.

**Table-II:** Technical data for Toris-K (HydroCone) contact lens.

Total diameter	12.00 → 17.00 mm
Base curve	7.00 → 10.80 mm
Sphere	- 40.00 → +40.00 dpt
Cylinder	- 0.25 → - 8.00 dpt
Axis	00 → 1800
Addition	+0.50 → +4.00 dpt
Flattening	K12 + K34 ++
Optimized centre thickness	Standard K12 = 0.42mm, K34 = 0.52 mm
Range of thickness	0.35 → 0.59 mm

This shows the success of a soft silicon-hydrogel contact lens, which is called Toris-K, in the management of keratoconus and traumatic keratopathy. It might provide a remarkable increase in visual acuity and be a good treatment option especially for the patients who are unsuitable for RGPCL. Further prospective, randomized, and controlled studies are needed to confirm this success.
